# The FLT3 Inhibitor Quizartinib Inhibits ABCG2 at Pharmacologically Relevant Concentrations, with Implications for Both Chemosensitization and Adverse Drug Interactions

**DOI:** 10.1371/journal.pone.0071266

**Published:** 2013-08-14

**Authors:** Jasjeet Bhullar, Karthika Natarajan, Suneet Shukla, Trevor J. Mathias, Mariola Sadowska, Suresh V. Ambudkar, Maria R. Baer

**Affiliations:** 1 Greenebaum Cancer Center, University of Maryland, Baltimore, Maryland, United States of America; 2 Laboratory of Cell Biology, National Cancer Institute, National Institutes of Health, Bethesda, Maryland, United States of America; 3 Department of Medicine, University of Maryland School of Medicine, Baltimore, Maryland, United States of America; Hungarian Academy of Sciences, Hungary

## Abstract

The oral second-generation bis-aryl urea fms-like tyrosine kinase 3 (FLT3) inhibitor quizartinib (AC220) has favorable kinase selectivity and pharmacokinetics. It inhibits mutant and wild-type FLT3 *in vivo* at 0.1 and 0.5 µM, respectively, and has shown favorable activity and tolerability in phase I and II trials in acute myeloid leukemia, with QT prolongation as the dose-limiting toxicity. Co-administration with chemotherapy is planned. We characterized interactions of quizartinib with the ATP-binding cassette (ABC) proteins ABCB1 (P-glycoprotein) and ABCG2 (breast cancer resistance protein). Its effects on uptake of fluorescent substrates and apoptosis were measured by flow cytometry, binding to ABCB1 and ABCG2 drug-binding sites by effects on [^125^I]iodoarylazidoprazosin ([^125^I]-IAAP) photolabeling and ATPase activity, and cell viability by the WST-1 colorimetric assay. Quizartinib inhibited transport of fluorescent ABCG2 and ABCB1 substrates in ABCG2- and ABCB1-overexpressing cells in a concentration-dependent manner, from 0.1 to 5 µM and from 0.5 to 10 µM, respectively, and inhibited [^125^I]-IAAP photolabeling of ABCG2 and ABCB1 with IC_50_ values of 0.07 and 3.3 µM, respectively. Quizartinib at higher concentrations decreased ABCG2, but not ABCB1, ATPase activity. Co-incubation with quizartinib at 0.1 to 1 µM sensitized ABCG2-overexpressing K562/ABCG2 and 8226/MR20 cells to ABCG2 substrate chemotherapy drugs in a concentration-dependent manner in cell viability and apoptosis assays. Additionally, quizartinib increased cellular uptake of the ABCG2 substrate fluoroquinolone antibiotic ciprofloxacin, which also prolongs the QT interval, in a concentration-dependent manner, predicting altered ciprofloxacin pharmacokinetics and pharmacodynamics when co-administered with quizartinib. Thus quizartinib inhibits ABCG2 at pharmacologically relevant concentrations, with implications for both chemosensitization and adverse drug interactions. These interactions should be considered in the design of treatment regimens combining quizartinib and chemotherapy drugs and in choice of concomitant medications to be administered with quizartinib.

## Introduction

The receptor tyrosine kinase fms-like tyrosine kinase 3 (FLT3) is expressed at high levels on malignant blasts in 70% to 100% of cases of acute myeloid leukemia (AML) [1], [2] and is mutated, most commonly by internal tandem duplication (ITD), in 20 to 30 percent of AML cases in different series [3]–[7]. FLT3-ITD mutations result in constitutive FLT3 signaling and, clinically, are associated with short disease-free survival (DFS) following chemotherapy [3]–[7]. FLT3 signaling may also be activated in AML cells by autocrine stimulation by FLT3 ligand [8].

Diverse kinase inhibitors inhibit signaling by both FLT3-ITD and wild-type FLT3. However first-generation inhibitors, including lestaurtinib, midostaurin, tandutinib sorafenib and sunitinib, lack optimal potency, selectivity and pharmacokinetic properties, resulting in limited activity and/or problematic toxicities, and have produced limited single-agent therapeutic benefit, mainly consisting of transient decreases in blasts [9]–[11]. The single randomized trial of a first-generation FLT3 inhibitor, lestaurtinib, in conjunction with chemotherapy reported to date did not demonstrate clinical benefit [12].

The second-generation bis-aryl urea FLT3 inhibitor quizartinib (AC220) has excellent kinase selectivity and pharmacokinetic properties [13] inhibits FLT3-ITD and wild-type FLT3 at 0.1 and 0.5 µM, respectively, *in vivo* [14] and has shown favorable tolerability and single-agent activity in phase I and II trials [15]–[17]. Of note, the dose-limiting toxicity of quizartinib is prolongation of the QT interval, which occurred in 38% and 6% of patients receiving continuous daily doses of 300 and 200 mg, respectively [15]. Following completion of early-phase clinical testing, quizartinib will be tested in combination with chemotherapy.

The ATP-binding cassette (ABC) proteins ABCB1 [P-glycoprotein (Pgp); MDR1] [18] and ABCG2 [breast cancer resistance protein (BCRP); mitoxantrone resistance protein (MXR)] [19] are drug efflux proteins that are frequently expressed on AML cells. Their substrates include anthracyclines (for ABCB1), mitoxantrone (for ABCG2) and other drugs used to treat AML, and their expression on AML cells is associated with inferior treatment outcomes [20], [21]. Co-administration of inhibitors of ABCB1 and ABCG2 drug efflux activity has the potential to sensitize AML cells to chemotherapy drugs that are substrates of these proteins. Unfortunately, however, clinical trials of ABCB1 inhibitors did not in fact demonstrate clinical benefit [22]–[26]. One of the possible reasons is lack of inhibition of ABCG2 [27], [28].

The first-generation FLT3 inhibitors, including midostaurin, lestaurtinib, tandutinib, sorafenib and sunitinib, are substrates and/or inhibitors of ABCB1 and ABCG2 [29]–[33]. Of note, in one recent study a significant positive correlation was found between FLT3-ITD and ABCG2 overexpression, and DFS was shortest in patients with AML with both FLT3-ITD and ABCG2 overexpression [34]. These data suggest that co-inhibition of FLT3 and of ABCG2 might be beneficial.

Since neutropenia caused by disease and/or therapy is frequent in AML patients, antibiotics and antifungals are commonly co-administered with AML therapy. Some of the antibiotic and antifungal agents prescribed to AML patients are ABCG2 and/or ABCB1 substrates and may also prolong the QT interval. Co-administration of drugs and FLT3 inhibitors with ABCG2 and/or ABCB1 interactions may alter pharmacokinetics and/or pharmacodynamics of either or both drugs.

We sought to characterize interactions of quizartinib with ABCB1 and ABCG2. Inhibition of these transport proteins on the surface of AML cells by quizartinib would result in sensitization to ABC protein substrate chemotherapy drugs, and co-inhibition of FLT3 and ABCG2 could improve outcomes. However, quizartinib inhibition of ABCG2 on intestinal mucosal cells [35] could result in increased absorption and altered pharmacokinetics of co-administered therapeutic agents that are ABC protein substrates, including drugs that cause QT prolongation, with consequent potential for enhancing cardiotoxicity.

## Materials and Methods

### Cell Lines

**Figure 1:**
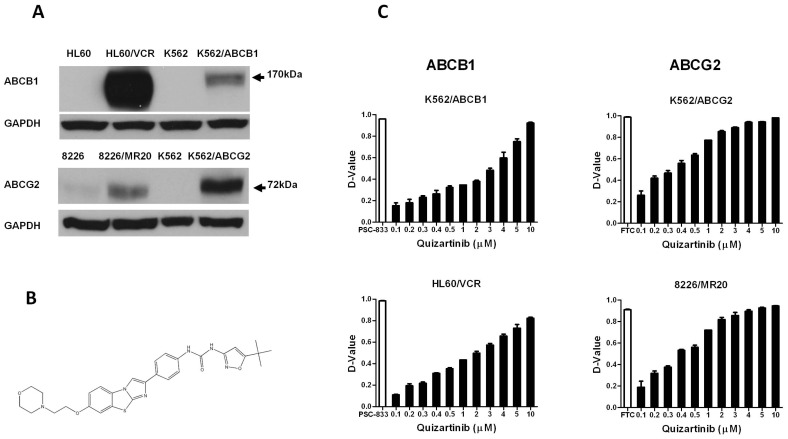
Quizartinib enhances uptake of substrates of ABCG2 and ABCB1 in a concentration-dependent manner in cells overexpressing these proteins. (**A**) ABCB1 and ABCG2 expression measured by immunoblotting in resistant and parental cell lines. (**B**) Quizartinib chemical structure. (**C**) Quizartinib effect on substrate transport by ABCB1 and ABCG2 was measured by comparing cellular fluorescence measured by flow cytometry after uptake of fluorescent substrates of these proteins, DiOC_2_(3) and pheophorbide A (PhA), respectively, in the presence and absence of quizartinib in HL60/VCR, K562/ABCB1 cells overexpressing ABCB1 and 8226/MR20 and K562/ABCG2 cells overexpressing ABCG2, with the ABCB1 modulator PSC-833 and ABCG2 modulator fumitremorgin C (FTC) as positive controls. Each bar represents the mean ± SEM of triplicate experiments. D-value is the Kolmogorov-Smirnov statistic and values ≥0.2 are considered significant.

Vincristine-selected HL60/VCR cells, [36] overexpressing ABCB1, were obtained from Dr. Ahmad R. Safa, Indiana University, Indianapolis, IN, and mitoxantrone-selected 8226/MR20 myeloma cells, [37] overexpressing wild-type ABCG2, with R482, [38], [39] from Dr. William Dalton, Moffitt Cancer Center, Tampa, FL. HL60/VCR cells were maintained in drug-free RPMI 1640 medium with 10% fetal bovine serum (FBS) and 8226/MR20 cells in RPMI 1640 medium with 10% FBS and 20 nM mitoxantrone. Transfected K562 cells stably overexpressing ABCB1 [40] or wild-type ABCG2 [41] were kind gifts from Dr. Michael Gottesman, National Cancer Institute, Bethesda, MD and Dr. Yoshikazu Sugimoto, Kyoritsu University of Pharmacy, Tokyo, Japan, respectively. They were cultured in RPMI 1640, pH 7.4, supplemented with 10% FBS at 37°C in a humidified atmosphere containing 5% CO_2_. ABCB1 and ABCG2 expression in resistant and parental cell lines is shown in Figure 1A. MCF-7 FLV1000 were cultured in RPMI 1640 with 10% FBS and were maintained in 1 µg/mL flavopiridol, as described previously [42]. HL60 and K562 cells were obtained from the American Type Culture Collection (Manassas, VA).

### Expression of ABCB1 and ABCG2 in High-Five Insect Cells

Generation of recombinant baculovirus encoding ABCB1-His6 and His6-ABCG2 and infection of High-Five insect cells was carried out as described previously [43].

### Materials

Quizartinib (Figure 1B) was purchased from Selleck Chemicals, Houston, TX, and was stored at −20°C as a 100 mM stock solution in dimethyl sulfoxide. As previously described, [44] the fluorescent ABCB1 substrate 3,3′-diethyloxacarbocyanine iodide [DiOC_2_(3)] was purchased from Sigma-Aldrich (St Louis, MO), and the fluorescent ABCG2 substrate pheophorbide A (PhA) from Frontier Scientific (Logan, VT), the ABCB1 inhibitor, PSC-833 was obtained from Novartis Pharmaceutical Corporation (East Hanover, NJ), and the ABCG2 inhibitor fumitremorgin C (FTC) was purchased from Sigma-Aldrich. [^125^I]Iodoarylazidoprazosin (IAAP) (2200 Ci/mmol) was purchased from PerkinElmer Life and Analytical Sciences (Waltham, MA). The ABCG2 substrate chemotherapy drugs [19] mitoxantrone and topotecan were also purchased from Sigma-Aldrich. Fluorescein isothiocyanate (FITC)-conjugated annexin V and propidium iodide (PI) were purchased from Trevigen (Gaithersburg, MD). Cell Proliferation Reagent WST-1 was purchased from Roche Diagnostics (Indianapolis, IN). Ciprofloxacin was purchased from Enzo Life Sciences (Farmingdale, NY). Hyclone Hank’s Balanced Salt Solution (HBSS) was purchased from Thermo Fisher Scientific Inc (Logan, UT).

### Immunoblotting

Cells were lysed in RIPA buffer (Sigma-Aldrich) supplemented with protease and phosphatase inhibitor cocktails (Roche Applied Science, Indianapolis, IN), protein concentrations were measured using the BCA Protein Assay Kit (Thermo Fisher Scientific Inc Logan, UT) according to kit instructions, and immunoblotting was performed. Briefly, 20 µg of protein from each cell line were electrophoresed and transferred onto a PVDF membrane, and immunoblots were incubated with individual primary antibodies, including 1∶2000 dilution of rabbit anti-ABCB1 (Santa Cruz Biotechnology, Dallas, TX) or 1∶2000 dilution of mouse anti-ABCG2 (Kamiya Biomedical, Seattle, WA) overnight at 4°C or 1∶3000 dilution of mouse anti-glyceraldehyde-3-phosphate dehydrogenase (GAPDH) (Millipore, Billerica, MA) for one hour at room temperature, followed by incubation with 1∶10,000 horseradish peroxidase-conjugated goat anti-rabbit or anti-mouse (Santa Cruz) for one hour at room temperature.

### Uptake of Fluorescent ABC Protein Substrates

To measure the effect of quizartinib on uptake of fluorescent ABC protein substrates, HL60/VCR and K562/ABCB1 cells (1×10^6^) were incubated for 30 minutes at 37°C with DiOC_2_(3) (0.6 ng/ml) and quizartinib (0–10 µM) or PSC-883 (2.5 µM) as a positive control and 8226/MR20 and K562/ABCG2 cells with PhA (1 µM) and quizartinib (0–10 µM) or FTC (10 µM) as a positive control. Cells were then washed twice, resuspended in phosphate-buffered saline (PBS) and kept on ice until analysis, then acquired on a FACSCanto II flow cytometer (BD Biosciences, San Jose, CA) and analyzed using FlowJo software (Tree Star, Inc., Ashland, OR). Substrate content after uptake with and without modulator was compared using the Kolmogorov-Smirnov statistic, expressed as a D*-*value ranging from 0 (no difference) to 1 (no overlap) [45] with D-values ≥0.2 indicating significant modulation based on previous work [46].

### Isolation of Crude Membranes

Membranes from High-Five insect cells expressing ABCB1 and MCF-7 FLV1000 expressing ABCG2 were isolated using hypotonic lysis and differential centrifugation as described previously [47], [48].

### Photoaffinity Labeling of ABCB1 and ABCG2 with [^125^I]IAAP

High-Five insect cell membrane vesicles expressing ABCB1 (50–70 µg protein) and crude membranes from MCF-7/Flv1000 cells (30 µg protein) expressing ABCG2 protein were incubated with 0–30 µM quizartinib for 5 minutes at 21–23°C in 50 mM Tris-HCl, pH 7.5. [^125^I]-IAAP (2200 Ci/mmole), 3–6 nM, was added and incubation was continued for 5 additional minutes under subdued light. Photoaffinity labeling of ABCB1 and ABCG2 with [^125^I] iodoarylazidoprazosin ([^125^I]-IAAP) was measured as previously described [48], [49].

### ABCB1 and ABCG2 ATPase Assay

The vanadate (Vi)- and beryllium fluoride (BeFx)-sensitive ATPase activity of ABCB1 and ABCG2 expressed in the membrane vesicles of High-Five insect cells in the presence of the indicated concentrations of quizartinib was measured as previously described [49], [50].

### Cell Viability Assay

Viability of drug-treated cells was evaluated using the WST-1 assay, as described previously [44]. Briefly, log-phase cells were seeded at 1×10^3^ in 100 µL of complete medium per well in 96-well tissue culture plates and incubated with quizartinib (0–10 µM) or chemotherapy drugs at a range of concentrations at 37°C in 5% CO_2_ for 96 hours. 10 µL of WST-1 reagent was then added to each well, and incubation was continued for 2 to 4 additional hours and the color developed was quantified according to the manufacturer’s instructions. Each experiment was performed in triplicate at least three times.

### Curve Shift Assay

The effect of quizartinib on sensitivity of ABCG2-overexpressing cell lines and their respective parental cells to ABCG2 substrate chemotherapy drugs (mitoxantrone, topotecan) was evaluated in cell viability assays. Briefly, cells were plated with chemotherapy drugs at a range of concentrations in the presence and absence of 0.1, 0.5 and 1 µM quizartinib and the established ABCG2 inhibitor FTC at 10 µM. Cell viability was measured with the WST-1 colorimetric assay, as described above. Resistance-modifying factors (RMF) were calculated as the ratios of IC_50_ values in the absence and presence of quizartinib at of 0.1, 0.5 or 1 µM or FTC at 10 µM. FTC exhibited some cytotoxicity toward K562 and K562/ABCG2 cells, so the data were normalized to DMSO control.

### Measurement of Apoptosis

8226/MR20 and K562/ABCG2 cells, overexpressing ABCG2, were incubated with mitoxantrone or topotecan at concentrations at or above their IC_50_s in cell viability assays for 48 hours in the presence and absence of quizartinib at a range of concentrations, and apoptosis and necrosis were measured by staining with annexin V-FITC and PI (Trevigen, Gaithersburg, MD) as previously described [44].

### Fluoroquinolone Uptake Assay

Fluoroquinolone antibiotics are ABCG2 substrates [51]. We studied the effect of quizartinib on uptake of the fluoroquinolone antibiotic ciprofloxacin in K562/ABCG2 and 8226/MR20 cells. Ciprofloxacin was measured by fluorescence spectrophotometry using a published method [52] with minor modifications. Briefly, 5x10^6^ cells were incubated with ciprofloxacin HCL (25 µg/ml) at 37°C for 30 mins in the presence of the ABCG2 inhibitor FTC at 10 µM or quizartinib at concentrations of 0.1 to 10 µM. The cells were then pelleted by centrifugation at 1400 rpm for 4 minutes, washed once with HBSS, resuspended in 200 µL HBSS and lysed by a quick freeze-thaw process (freezing at −80°C for 20 minutes and thawing to room temperature). The cell lysates were clarified by centrifugation at 8000×g for 10 minutes at room temperature. 100 µL of the resultant supernatant were then acidified by addition of 2 µL 0.1N HCL and flourescence was measured on a Synergy HT Multi-Mode Microplate Reader (BioTek, Winooski, VT) and Gen5™ software in the time-resolved fluorescence mode with excitation and emission wavelengths of 278 and 460 nm, respectively. The results were plotted graphically after correcting for background fluorescence.

### Statistical Analysis

IC_50_ values were calculated by the least square fit of dose-response inhibition in a non-linear regression model. Statistical analysis was performed using GraphPad Prism 5 software (GraphPad Software, Inc., La Jolla, CA). Percentages of apoptotic cells were compared by two-way ANOVA with post-hoc Bonferroni testing. Statistical significance was defined by p-values <0.05. For the fluoroquinolone uptake assay, fluorescence in the presence of quizartinib at each concentration was compared with fluorescence in the absence of quizartinib using one way ANOVA with Dunnett’s post hoc test.

## Results

### Quizartinib Inhibits Substrate Transport by ABCG2 and ABCB1 in a Concentration-Dependent Manner

Quizartinib inhibited transport of the fluorescent ABCB1 substrate DiOC_2_(3) in ABCB1-overexpressing drug-selected HL60/VCR and transfected K562/ABCB1 cells and transport of the fluorescent ABCG2 substrate pheophorbide A in ABCG2-overexpressing drug-selected 8226/MR20 and transfected K562/ABCG2 cells, in a concentration-dependent manner (Figure 1C). Of note, Quizartinib inhibition of transport occurred at lower concentrations for ABCG2 than for ABCB1, with 50% inhibition in relation to positive controls (FTC for ABCG2 and PSC-833 for ABCG2) at concentrations of approximately 0.5 µM for ABCG2, compared to 3 µM for ABCB1. The concentrations at which quizartinib inhibits ABCG2-mediated transport are in the range of those required for inhibition of FLT3-ITD and wild-type FLT3 *in vivo,* 0.1 and 0.5 µM, which correspond to doses of 12 mg and 60 mg, respectively, [14] whereas inhibition of ABCB1-mediated transport appeared to occur at concentrations above those targeted clinically.

### Quizartinib Inhibits [^125^I]-IAAP Photolabeling of ABCG2 and ABCB1

**Figure 2:**
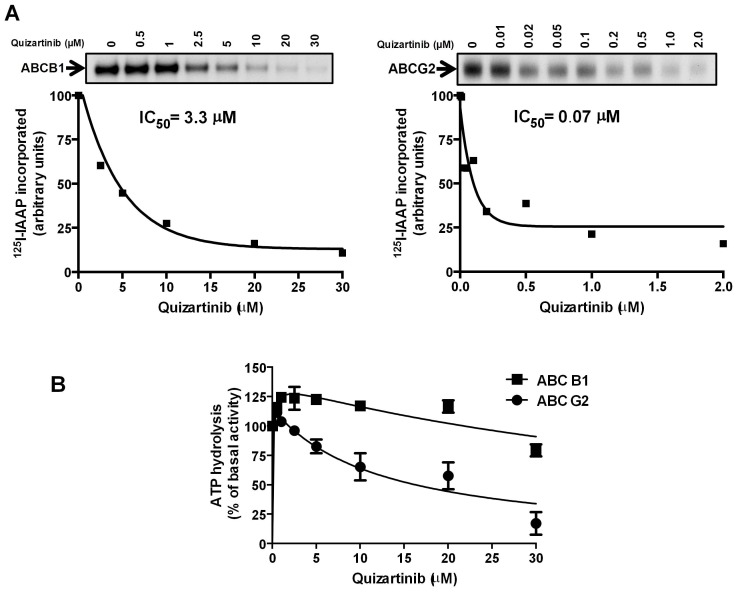
Quizartinib decreased [^125^I]-IAAP photolabeling of both ABCB1 and ABCG2 but inhibited ATPase activity of only ABCG2 at high concentrations. Crude membranes from High-Five insect cells expressing ABCB1 and MCF-7 FLV1000 cells expressing ABCG2 were incubated with 0–30 µM quizartinib for 5 minutes at 21–23°C in 50 mM Tris-HCl, pH 7.5 and 3–6 nM [^125^I]-IAAP (2200 Ci/mmole) was added, followed by processing as described in Materials and Methods. (**A**) Representative autoradiograms from one experiment are shown in the upper panels; similar results were obtained in two additional experiments. In the lower panels, incorporation of [^125^I]-IAAP (from autoradiogram, Y-axis) into the ABCB1 and ABCG2 bands was plotted as a function of quizartinib concentration (X-axis). Quizartinib inhibited [^125^I]-IAAP binding to ABCB1 and ABCG2 with IC_50_’s of 3.3 µM and 0.07 µM, respectively, and the latter correspond to a therapeutically relevant plasma concentration. Values are from a representative experiment among three independent experiments. (**B**) Crude membrane protein from High Five insect cells expressing ABCB1 or ABCG2 was incubated with quizartinib at a range of concentrations in the presence or absence of sodium orthovanadate or BeFx (beryllium sulfate and sodium fluoride), respectively, as described in Materials and Methods. The ATPase activity in the presence of the indicated concentrations of quizartinib was calculated as a percent of basal (no addition of drug). The mean and standard error values from three independent experiments for ABCB1 (filled squares) and ABCG2 (filled circles) are shown.

Since quizartinib inhibited substrate transport by ABCG2 and ABCB1 in a concentration-dependent manner, we sought to confirm that it interacted with established drug-binding sites of these transport proteins. To this end, we measured effects of quizartinib on photolabeling of ABCG2 and ABCB1 with [^125^I]-IAAP. Quizartinib was found to inhibit [^125^I]-IAAP photolabeling of ABCG2 and ABCB1 with IC_50_ values of 0.07 µM and 3.3 µM, respectively (Figure 2A). These data are consistent with binding of quizartinib to drug-binding sites on both proteins, with binding to ABCG2 at a lower concentration that to ABCB1, correlating with the effective concentrations for inhibition of drug transport (Figure 1C).

### Quizartinib Inhibits ABCG2, but not ABCB1, ATPase Activity

To further characterize the interactions between quizartinib and ABCG2 and ABCB1, we studied the effect of quizartinib on their ATPase activity (Figure 2B). Quizartinib inhibited ABCG2 ATPase activity in a concentration-dependent manner, but only at relatively high concentrations, in the range of 5 µM and above. This effect was similar to that of the established ABCG2 inhibitor FTC [53]. It should be noted that quizartinib at lower concentrations (0.05–2 µM) showed a small stimulatory effect on ATPase activity of both ABCB1 (∼23% stimulation) and ABCG2 (∼14% stimulation), which supports its interaction at the substrate-binding pocket, as is also shown in Figure 2A above, indicating that quizartinib behaves similarly to other established transported substrates of these transporters [54]–[56].

### Quizartinib Sensitizes ABCG2-overexpressing Cells to Substrate Chemotherapy Drugs in Cell Viability Assays

**Figure 3:**
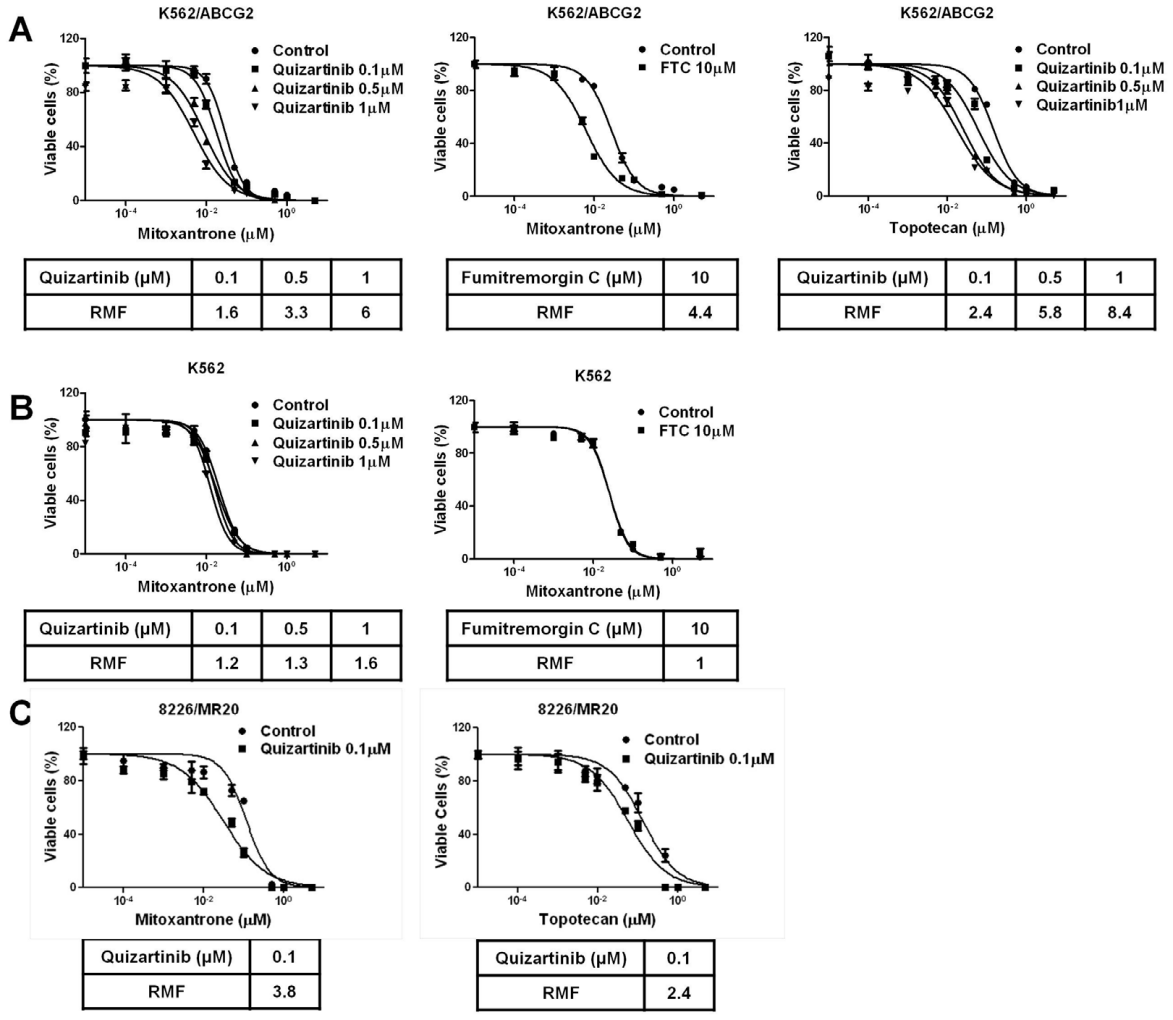
Quizartinib sensitizes resistant cells overexpressing ABCG2, but not parental cells, to cytotoxic effects of ABCG2 substrate chemotherapy drugs. (**A**) K562/ABCG2 cells, overexpressing ABCG2, were plated with mitoxantrone and topotecan at increasing concentrations in the absence and presence of quizartinib at 0.1, 0.5 and 1 µM and the established ABCG2 transport inhibitor fumitremorgin C at 10 µM for 96 hours and viable cells were measured using the WST-1 assay. Chemosensitization was quantified as the resistance modifying factor (RMF), or ratio of IC_50_ values in the absence and presence of quizartinib at each concentration. (**B**) Parental K562 cells were plated with mitoxantrone at increasing concentrations in the absence and presence of quizartinib at 0.1, 0.5 and 1 µM and the established ABCG2 transport inhibitor fumitremorgin C at 10 µM. (**C**) 8226/MR20 cells, expressing ABCG2, were plated with mitoxantrone and topotecan at increasing concentrations in the absence and presence of quizartinib at 0.1 µM only, as higher concentrations were cytotoxic.

Because quizartinib bound to ABCG2, but not ABCB1, and inhibited ABCG2, but not ABCB1, substrate transport at therapeutically relevant concentrations, we studied its effects in sensitizing cell lines with drug resistance mediated by ABCG2 to substrate chemotherapy drugs. Co-incubation with quizartinib at 0.1, 0.5 and 1 µM sensitized K562/ABCG2 cells 1.6-, 3.3- and 6-fold to mitoxantrone and 2.4-, 5.8- and 8.4-fold to topotecan at fixed concentrations in cell viability assays (Figure 3A), a chemosensitizing effect of equal magnitude to that of the established ABCG2 inhibitor FTC, which sensitized K562/ABCG2 4.4-fold to mitoxantrone at the standard concentration of 10 µM. In contrast, quizartinib did not sensitize parental K562 cells to mitoxantrone (Figure 3B). Co-incubation with 0.1 µM quizartinib also sensitized ABCG2-overexpressing 8226/MR20 cells 3.8-fold to mitoxantrone and 2.4-fold to topotecan (Figure 3C), while quizartinib was cytotoxic to this cell line at concentrations of 0.5 and 1 µM. Thus quizartinib at therapeutically relevant concentrations sensitizes cells with chemoresistance mediated by ABCG2, but not parental cells, to ABCG2 substrate chemotherapy drugs, and this effect was similar in magnitude to that of the established ABCG2 inhibitor FTC.

### Quizartinib Enhances Apoptosis Induction by ABCG2 Substrate Chemotherapy Drugs in ABCG2-overexpressing Cells

**Figure 4:**
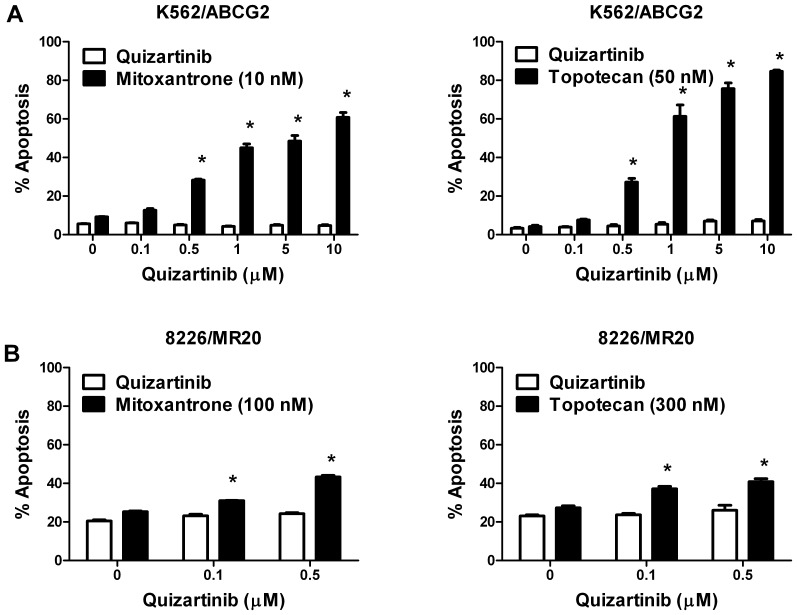
Quizartinib sensitizes resistant cells overexpressing ABCG2 to apoptosis induction by ABCG2 substrate chemotherapy drugs. K562/ABCG2 (**A**) and 8226/MR20 (**B**) cells overexpressing ABCG2 were incubated with the ABCG2 substrate chemotherapy drugs mitoxantrone and topotecan at fixed concentrations in the absence and presence of quizartinib at a range of concentrations for 48 hours. Apoptosis was measured by staining with annexin V-FITC and propidium iodide, detected by flow cytometry. Each bar represents the mean ± SEM percentages of apoptotic cells in triplicate experiments. *p<0.0001.

We also determined the effect of quizartinib on apoptosis induction by ABCG2 substrate chemotherapy drugs in ABCG2-overexpressing cells. Co-incubation with quizartinib at concentrations of 0.1, 0.5, 1, 5 and 10 µM increased apoptosis of K562/ABCG2 cells induced by the ABCG2 substrate chemotherapy drugs mitoxantrone and topotecan (p<0.001) at fixed concentrations (Figure 4). Similarly, co-incubation with quizartinib at concentrations of 0.1 and 0.5 µM increased apoptosis of 8226/MR20 cells induced by mitoxantrone and topotecan (p<0.001) at fixed concentrations, with quizartinib alone at higher concentrations inducing apoptosis of 8226/MR20 (data not shown).

### Quizartinib Increases Uptake of Fluoroquinolones in Cells Expressing ABCG2

**Figure 5:**
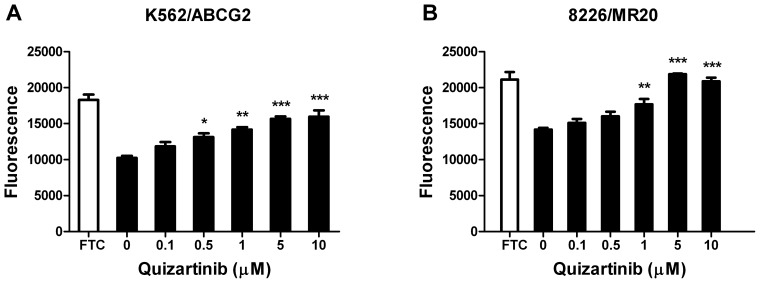
Quizartinib increases uptake of fluoroquinolones in cells expressing ABCG2. K562/ABCG2 (**A**) and 8226/MR20 (**B**) cells, overexpressing ABCG2, were incubated with ciprofloxacin at a fixed concentration (25µg/ml) in the absence and presence of quizartinib at a range of concentrations for 30 minutes, and with the ABCG2 transport inhibitor fumitremorgin C (FTC) as a positive control. Cellular fluorescence was measured on a microplate reader. *p<0.05, **p<0.01 and ***p<0.001.

We hypothesized that, in addition to chemosensitizing leukemia cells overexpressing ABCG2 to ABCG2 substrate drugs, quizartinib, which is orally administered, would inhibit ABCG2 on intestinal mucosal cells, and thus increase absorption of co-administered oral ABCG2 substrate drugs, including those with the potential to contribute to QT prolongation. One such class of drugs is the fluoroquinolone antibiotics, which are commonly administered to AML patients to prevent infections in the setting of neutropenia. To test the effect of quizartinib on transport of fluoroquinolone antibiotics, we incubated K562/ABCG2 and 8226/MR20 cells with ciprofloxacin at a fixed concentration of 25 µg/ml in the presence of quizartinib at increasing concentrations, and demonstrated that quizartinib increased ciprofloxacin accumulation in a concentration-dependent manner (Figure 5). Of note, quizartinib itself was not fluorescent at the concentrations studied (data not shown).

### K562/ABCB1 and K562/ABCG2 Cells Exhibit Collateral Sensitivity to Quizartinib

**Figure 6:**
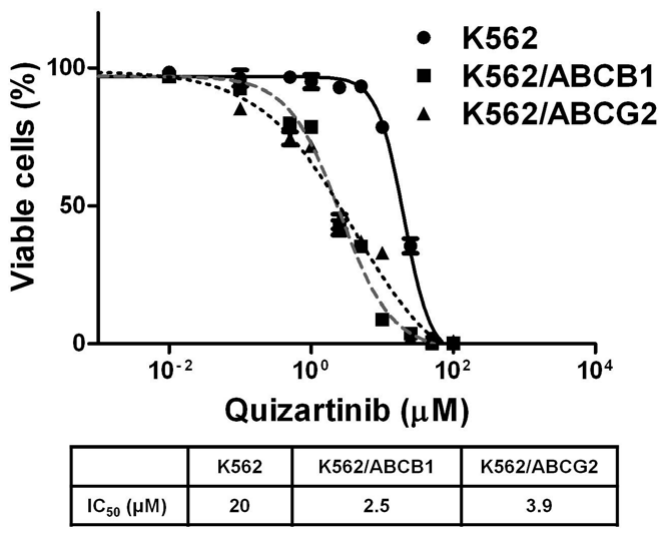
K562/ABCB1 and K562/ABCG2 cells exhibit collateral sensitivity to **quizartinib.** K562, K562/ABCB1 and K562/ABCG2 cells were plated in the presence of quizartinib in increasing concentrations for 96 hours and viable cells were measured using the WST-1 assay.

K562, K562/ABCB1 and K562/ABCG2 cells were cultured with quizartinib at a range of concentrations in a 96-hour cytotoxicity assay. IC_50_ values for quizartinib in K562, K562/ABCB1 and K562/ABCG2 were 20, 2.5 and 3.9 µM, respectively (Figure 6), indicating collateral sensitivity [57] of K562/ABCB1 and K562/ABCG2 cells to quizartinib.

## Discussion

We have demonstrated that quizartinib is a potent inhibitor of ABCG2-mediated drug transport at pharmacologically relevant concentrations. Quizartinib inhibition of ABCG2 should therefore sensitize ABCG2-overexpressing AML cells to co-administered ABCG2 substrate chemotherapy drugs. In addition, however, as a less desirable effect, quizartinib, which is orally administered, should inhibit ABCG2 on intestinal mucosal cells and thereby increase intestinal uptake and alter pharmacokinetics of orally co-administered ABCG2 substrate drugs, including those that prolong the QT interval.

The data from [^125^I]-IAAPbinding and ATPase assays indicate that quizartinib directly interacts at the drug-binding pocket of both ABCB1 and ABCG2. The fact that it is ∼5-fold more potent in inhibiting binding of [^125^I]-IAAP to ABCG2 than to ABCB1 indicates that it may have differences in binding affinity to the substrate-binding pockets of these transporters. It is known that some substrates, including other tyrosine kinase inhibitors, stimulate ATPase activity of ABCB1 and ABCG2 at lower concentrations, but inhibit ATPase activity at higher concentrations [54]–[56], [58], [59]. Taken together, the data in Figure 2 suggest that quizartinib may be transported by both ABCB1 and ABCG2 at lower concentrations. However, this conclusion needs to be validated by measuring the net efflux of quizartinib from polarized cells such as LLC-PK1 or MDCK expressing ABCB1 or ABCG2. This will help further in understanding the role of these transporters in altering quizartinib sensitivity or bioavailability.

Quizartinib was found to exhibit collateral sensitivity in K562/ABCB1 and K562/ABCG2 cells, in relation to parental K562 cells. Collateral sensitivity is an incompletely understood phenomenon, for which four possible mechanisms have been proposed: 1) production of reactive oxygen species via futile hydrolysis of ATP, 2) exploitation of energetic sensitivities, 3) extrusion of endogenous substrates that are essential for cell survival, or 4) perturbation of the plasma membrane [57]. The first and third of these mechanisms require direct interaction with the ABC protein, as appears to be the case for quizartinib with ABCB1 and ABCG2.

Chemotherapeutic agents that are used to treat AML and that are ABCG2 substrates include mitoxantrone [60], topotecan [60], flavopiridol [61] and the nucleoside analogs cladribine, clofarabine and fludarabine [62]. All of these drugs are currently in use or under investigation in diverse therapeutic regimens in AML [63]–[67]. Based on our data presented here, co-administration of quizartinib has the potential to chemosensitize AML cells to any of these drugs, and thus the potential to enhance their efficacy and/or allow their administration at lower doses, thereby decreasing toxicity.

A significant correlation was recently reported between presence of FLT3-ITD and ABCG2 overexpression in pre-treatment AML cells [34]. Moreover, DFS was significantly shorter in patients with both FLT3-ITD and ABCG2 overexpression. Of note, patients in this series were treated with a fludarabine-based chemotherapy regimen, and fludarabine is an ABCG2 substrate. Co-administration of quizartnib with chemotherapy and, particularly, with ABCG2 substrate chemotherapy drugs, has the potential to overcome the negative impact of both FLT3-ITD and ABCG2 overexpression.

The dose-limiting toxicity of quizartinib is QT prolongation, and it may be exacerbated by co-administration of other drugs that prolong the QT interval. These include, among others, fluoroquinolone antibiotics, phenothiazine antiemetics, methadone, and the antiarrhythmic agents quinidine, procainamide, sotalol, amiodarone and verapamil [68]. The fluoroquinolone antibiotics, with those in current use including ciprofloxacin, levofloxacin and moxifloxacin, are drugs that are commonly administered to AML patients and may therefore be commonly co-administered with quizartinib. The fluoroquinilones are ABCG2 substrates [51], and their intestinal absorption and pharmacokinetic profile may therefore be altered when they are co-administered with quizartinib. Indeed we demonstrated that quizartinib increased accumulation of ciprofloxacin in a concentration-dependent manner in cells overexpressing ABCG2.

An additional potential consequence of ABCG2 inhibition in patients with AML is impact on the risk of hyperuricemia and gout. ABCG2 has been found to be a urate efflux transporter, with increased incidence of both hyperuricemia and gout in association with the Q141K ABCG2 single nucleoside polymorphism, which results in decreased urate transport [69]. It is therefore logical to infer that inhibition of ABCG2 function by agents such as quizartinib has the potential to increase uric acid levels, which should be mitigated by co-administration of a urate-lowering agent.

Finally, while clinically targeted plasma levels of quizartinib are below those needed for inhibition of ABCB1-mediated transport of chemotherapy drugs in AML cells, levels in the gastrointestinal tract are likely to be sufficient to inhibit ABCB1-mediated intestinal drug transport [35] and thus increase absorption of co-administered ABCB1 substrate drugs, including those that have the potential to prolong the QT interval [70].

We have shown that the second-generation bis-aryl urea FLT3 inhibitor quizartinib is a potent inhibitor of drug transport by ABCG2 at clinically targeted concentrations and thus may sensitize AML cells expressing ABCG2 to ABCG2 substrate chemotherapy drugs. It may have particular impact in the prognostically unfavorable subset of patients whose AML cells exhibit both FLT3-ITD and ABCG2 overexpression. Quizartinib also likely increases intestinal uptake and alters the pharmacokinetic profile of orally co-administered ABCG2 substrate drugs, including those that prolong the QT interval, such as fluoroquinolone antibiotics. Quizartinib inhibits drug transport by ABCB1 at higher concentrations, and is unlikely to chemosensitize, but likely increases intestinal uptake of orally co-administered ABCB1 substrate drugs, which include agents that prolong the QT interval. These interactions should be considered in the design of combination regimens incorporating quizartinib and chemotherapeutic agents and in choice of concomitant medications to be administered with quizartinib.
